# Effects of *CYP3A4* Polymorphisms on Drug Addiction Risk Among the Chinese Han Population

**DOI:** 10.3389/fpubh.2019.00315

**Published:** 2019-11-19

**Authors:** Li Wang, Mei Bai, Tianbo Jin, Jianwen Zheng, Yuhe Wang, Yongjun He, Dongya Yuan, Xue He

**Affiliations:** ^1^Key Laboratory of Molecular Mechanism and Intervention Research for Plateau Diseases of Tibet Autonomous Region, School of Medicine, Xizang Minzu University, Xianyang, China; ^2^School of Basic Medical Sciences, Xizang Minzu University, Xianyang, China; ^3^Department of Neurology, Affiliated Hospital of Xizang Minzu University, Xianyang, China; ^4^Department of Clinical Laboratory, Affiliated Hospital of Xizang Minzu University, Xianyang, China

**Keywords:** *CYP3A4*, drug addiction, case-control study, polymorphisms, Chinese Han population

## Abstract

**Background:** *Cytochrome P450 3A4* (*CYP3A4*) regulates pharmacokinetic and pharmacodynamic interactions during the process of drug absorption and metabolism, suggesting *CYP3A4* plays an important role in drug addiction. However, the association between *CYP3A4* polymorphisms and drug addiction risk is still not clear.

**Methods:** This case-control study included 504 drug addicts and 501 healthy controls from Xi'an, China. Four single nucleotide polymorphisms (SNP) in *CYP3A4* (rs3735451, rs4646440, rs35564277, and rs4646437) were genotyped by Agena MassARRAY platform. After adjusting by age and gender, we calculated odd ratios (OR) and 95% confidence intervals (CI) by logistic regression to estimate the association between *CYP3A4* polymorphisms and drug addiction risk.

**Results:** We found rs4646440 and rs4646437 were associated with decreased risk of drug addiction in codominant (rs4646440: *OR* = 0.41, 95%CI = 0.19–0.92, *p* = 0.030; rs4646437: *OR* = 0.19, 95%CI = 0.04–0.87, *p* = 0.032) and recessive (rs4646440: *OR* = 0.41, 95%CI = 0.19–0.91, *p* = 0.028; rs4646437: *OR* = 0.20, 95%CI = 0.04–0.90, *p* = 0.036) models. Rs3735451 and rs4646437 were associated with drug addiction risk in the subgroup of middle-aged people (44 < age ≤ 59) and elderly people (age ≥ 60), individually. For men, rs3735451, rs4646440, and rs4646437 had strong relationship with decreased risk of drug addiction (*p* < 0.05). The effects of rs3735451 on drug addiction risk were related to drug-using time (*p* < 0.05). We also observed one block (rs4646440 and rs35564277) in haplotype analysis.

**Conclusion:** *CYP3A4* polymorphisms were associated with drug addiction risk among the Chinese Han population.

## Introduction

Drug addiction is a chronic relapse disorder characterized by compulsive drug seeking tendencies and usage, paired with substantial morbidity and mortality (1, 2).Worldwide, 99,000–253,000 deaths a year are attributed to drug addiction (3). It is reported that the number of drug users reached nearly 2.96 million until 2014 in China (4). Epidemiological data showed that women had lower rates of drug use than men (5). Drug addiction is influenced by many factors, including environmental, mental, and genetic factors (6, 7). Accumulating studies had proved that gene variety leads to drug addiction accounting for ~50% (6, 8). During the drug addiction, age and sex differences are obvious in clinical and preclinical studies (9). Recently, drug-metabolizing enzyme had been addressed as a major target for drug addiction (10).

Cytochrome P450 (CYP) enzymes are monooxygenases that catalyze many reactions involved in the metabolism of drug, environmental contaminants, steroids and other lipids (11). The CYP3A4 enzyme is the most important drug-metabolizing P450s in the liver, which encoded by *CYP3A4* gene (12). CYP3A4 enzyme is mainly responsible for methadone metabolism and hence uses for the treatment of drug addiction (13). *CYP3A4* polymorphisms were also significantly associated with sedation side effects caused by methadone and several clinical conditions (ischemic stroke and epilepsy) (14–16). Previous study showed *CYP3A4* rs2242480 was significantly associated with drug addiction in Xi'an Han Chinese population (17), and CYP3A4^*^4 allele was related to an increase in the lipid-lowering effects of simvastatin by decreasing *CYP3A4* activity (18). In the *CYP3A4* gene, rs4646440, and rs4646437 were the most common polymorphisms. Studies revealed that rs4646440 had strong relationship with withdrawal symptoms and adverse reactions in methadone maintenance patients (14). Rs4646437 had effects on the risk of many diseases, such as hypertension, human immunodeficiency virus (HIV) and some cancers (19–21). However, the study of association between *CYP3A4* polymorphisms and drug addiction risk among the Chinese Han population is scarcely.

To assess the effects of *CYP3A4* polymorphisms on drug addiction risk, we conducted a case-control study to explore the association of *CYP3A4* polymorphisms (rs3735451, rs4646440, rs35564277, and rs4646437) and drug addiction risk among the Chinese Han population.

## Methods

### Study Participants

Between 2016 and 2018, 504 drug addicts were recruited from Xi'an addiction treatment center and 501 healthy controls were randomly selected from Xi'an medical examination center. All cases had daily uses of narcotics (including hemp, opium, and cocaine) for 1 or more years and had a clinical characteristics of drug addiction, which confirmed by urine tests. The patients with seizure disorder, psychotic or severe medical illness were excluded. The control subjects were healthy individuals assessed by the Addiction Severity Index (22, 23) and people had endocrine, metabolic, nutritional or severe diseases were excluded. All participants were over the age of 18 and signed informed consents before study. We obtained written informed consents from all participants. Our study was approved by the ethic committee of Clinical Research Ethics of Northwest University.

### Genotyping

Combined previous studies, four polymorphisms (rs3735451, rs4646440, rs35564277, and rs4646437) with minor allele frequencies >5% in the Chinese Han Beijing population were selected. Genomic DNA was extracted from whole blood samples by GoldMag–Mini Purification Kit (GoldMag Co. Ltd. Xi'an, China) and was quantified by DU530 UV/VIS spectrophotometer (Beckman Instruments, Fullerton, CA, USA). Agena MassARRAY Assay Design 3.0 software was used to design primers in this study (Table 1). Genotyping was performed by the standard protocol from the Agena MassARRAY RS1000 manufacturer, data were managed and analyzed using the Agena Typer 4.0 Software (24).

**Table 1:** Primers used in the study.

**SNP**	**1st-PCRP**	**2nd-PCRP**	**UEP_DIR**	**UEP_SEQ**
rs3735451	ACGTTGGATGCAAAGTGAGTGAGACACTCC	ACGTTGGATGTACTGCATTTTTTTTGCCC	R	ccccTTTGCCCATTACTCCAT
rs4646440	ACGTTGGATGATGCTAAGGATTTCAGTCCC	ACGTTGGATGCCAACTATGATGTGTGGAGG	F	cccgcTGTGTGGAGGAGTTATGAAGT
rs35564277	ACGTTGGATGGGCCCAACTTGTAATCATAG	ACGTTGGATGTGGACAAAAAGCTAGATGAG	F	CAAAAAGCTAGATGAGTGGTAA
rs4646437	ACGTTGGATGCTTCAAAAGATGCACAAGGG	ACGTTGGATGAGGGCAGGTCTATGCATAAG	F	ctgaAGGTCTATGCATAAGGAGCACC

*SNP, single nucleotide polymorphism*.

### Statistical Analysis

We used Microsoft Excel and SPSS 18.0 (SPSS, Chicago, IL) to conduct statistical analysis. All *p*-values were two-sided and *p* < 0.05 was regard as statistical significance. The Hardy-Weinberg equilibrium (HWE) for each single nucleotide polymorphism (SNP) in controls was evaluated by Chi-square test. The categorical and continuous variables were assessed using Chi-square test and *t*-test, individually. After adjusted by age and gender, the association of *CYP3A4* polymorphisms and drug addiction risk was estimated using logistic regression analysis by calculating odd ratio (OR) and 95% confidence intervals (CI). Genetic models (codominant, dominant, recessive, and additive) was performed on PLINK software. Linkage disequilibrium (LD) and haplotype construction were conducted by Haploview software (version 4.2) (25).

## Results

The characteristics of participants were presented in Table 2. Our study included 504 cases (448 men, 56 women) and 501 controls (447 men, 54 women). The mean ages of cases and controls were 48.46 ± 6.88 and 48.67 ± 8.01, respectively. There were no significant differences in age and gender between two groups (age: *p* = 0.308, gender: *p* = 0.920). For all cases, 158 (31%) had drug addiction more than 16 years, 144 (28%) had drug addiction equal or <16 years, the other people (205, 41%) did not have the information of drug-using time

**Table 2:** Characteristics of participants in this study.

**Variable**	**Cases (*N* = 504)**	**Controls (*N* = 501)**	***p***
Age (Years old)	48.46 ± 6.88	48.67 ± 8.01	0.308
≤44	130 (26%)	146 (29%)	
45–59	351 (70%)	317 (74%)	
≥60	23 (4%)	38 (7%)	
Gender			0.920
Man	448 (89%)	447 (89%)	
Woman	56 (11%)	54 (11%)	
Drug-using time (Years)			
>16	158 (31%)		
≤16	141 (28%)		
Absence	205 (41%)		

*SNP, single nucleotide polymorphism*.

As shown in Table 3, the MAFs of four SNPs in two groups were listed, and all SNPs were in HWE (*p* > 0.05). The association between *CYP3A4* polymorphisms and drug addiction risk in allele model was also shown in Table 3. Compared with GG genotype, the individuals with rs4646440 AA genotype had significantly decreased risk of drug addiction (*OR* = 0.41, 95%CI = 0.19–0.92, *p* = 0.030). In recessive model, rs4646440 had strong relationship with drug addiction risk (*OR* = 0.41, 95%CI = 0.19–0.91, *p* = 0.028). Additionally, rs4646437 was significantly associated with drug addiction risk in codominant (*OR* = 0.19, 95%CI = 0.04–0.87, *p* = 0.032) and recessive (*OR* = 0.20, 95%CI = 0.04–0.90, *p* = 0.036) models.

**Table 3:** The association of *CYP3A4* polymorphisms and drug addiction risk.

**SNP**	**Position**	**MAF in cases**	**MAF in controls**	**HWE- *p***	**Model**	**Allele/Genotype**	**OR(95%CI)**	***p***
rs3735451	Chr7:99758352	0.199	0.216	0.674	Allele	C/T	0.89(0.73–1.08)	0.227
					Codominant	CC/TT	0.69(0.42–1.12)	0.134
						CT/TT	0.95(0.73–1.23)	0.687
					Dominant	CC-CT/TT	0.90(0.71–1.16)	0.426
					Recessive	CC/CT-TT	0.71(0.44–1.13)	0.149
					Additive		0.88(0.72–1.08)	0.214
rs4646440	Chr7:99763247	0.059	0.072	0.599	Allele	A/G	0.90(0.73–1.12)	0.342
					Codominant	AA/GG	0.41(0.19–0.92)	**0.030**
						AG/GG	1.02(0.79–1.32)	0.885
					Dominant	AA-AG/GG	0.95(0.74–1.23)	0.722
					Recessive	AA/AG-GG	0.41(0.19–0.91)	**0.028**
					Additive		0.89(0.71–1.12)	0.309
rs35564277	Chr7:99764813	0.127	0.873	0.734	Allele	C/T	0.80(0.56–1.14)	0.222
					Codominant	CC/TT	–	–
						CT/TT	0.87(0.59–1.26)	0.450
					Dominant	CC-CT/TT	0.83(0.57–1.20)	0.319
					Recessive	CC/CT-TT	–	–
					Additive		0.80(0.56–1.14)	0.216
rs4646437	Chr7:99767460	0.526	0.498	0.609	Allele	A/G	0.80(0.62–1.03)	0.085
					Codominant	AA/GG	0.19(0.04–0.87)	**0.032**
						AG/GG	0.87(0.66–1.16)	0.355
					Dominant	AA-AG/GG	0.83(0.63–1.09)	0.184
					Recessive	AA/AG-GG	0.20(0.04–0.90)	**0.036**
					Additive		0.79(0.61–1.03)	0.077

*SNP, single nucleotide polymorphism; MAF, minor allele frequency; HWE, Hardy-Weinberg Equilibrium; OR, odds ratio; CI, confidence interval. Bold data means significant difference (p < 0.05). –, No data*.

Furthermore, we performed stratified analysis of association between *CYP3A4* polymorphisms and drug addiction risk (Table 4). Rs4646437 had a strong relationship with drug addiction among middle-aged people (44 <Age ≤ 59) in allele (*OR* = 0.73, 95%CI = 0.54–1.00, *p* = 0.046) and additive (*OR* = 0.72, 95%CI = 0.52–0.99, *p* = 0.044) models. For the individuals equal or more than 60 years old, rs4646440 was related to increased drug addiction risk in allele model (*OR* = 3.83, 95%CI = 1.22–12.07, *p* = 0.016). In the subgroup of man, rs3735451, rs4646440, and rs4646437 were related to decreased drug addiction risk in codominant (rs3735451: *OR* = 0.39, 95%CI = 0.16–0.97, *p* = 0.042; rs4646440: *OR* = 0.39, 95%CI = 0.16–0.97, *p* = 0.042; rs4646437: *OR* = 0.12, 95%CI = 0.01–0.97, *p* = 0.046) and recessive (rs4646440: *OR* = 0.40, 95%CI = 0.16–0.97, *p* = 0.043) models. Moreover, the effects of rs3735451 on drug addiction were related to drug-using time (codominant: *OR* = 0.29, 95%CI = 0.10–0.81, *p* = 0.018; recessive: *OR* = 0.28, 95%CI = 0.10–0.79, *p* = 0.015).

**Table 4:** Stratified analysis of association between *CYP3A4* polymorphisms and drug addiction risk.

**SNP**	**Model**	**Age** **≤** **44**		**44** ** <Age** **≤** **59**		**Age** **≥** **60**		**Man**		**Drug-using time**	
		**OR(95%CI)**	***p***	**OR(95%CI)**	***p***	**OR(95%CI)**	***p***	**OR(95%CI)**	***p***	**OR(95%CI)**	***p***
rs3735451	Allele	0.84 (0.59–1.22)	0.365	0.92 (0.72–1.16)	0.467	0.81 (0.36–1.85)	0.621	0.89 (0.73–1.09)	0.273	0.76 (0.53–1.09)	0.140
	Codominant	0.46 (0.18–1.18)	0.106	0.82 (0.44–1.54)	0.544	1.13 (0.19–6.61)	0.892	0.39 (0.16–0.97)	**0.042**	0.29 (0.10–0.81)	**0.018**
		1.12 (0.68–1.85)	0.649	0.92 (0.67–1.27)	0.628	0.46 (0.13–1.60)	0.222	0.97 (0.73–1.28)	0.822	1.02 (0.62–1.67)	0.943
	Dominant	0.97 (0.60–1.57)	0.913	0.91 (0.67–1.24)	0.548	0.59 (0.19–1.78)	0.344	0.90 (0.69–1.17)	0.420	0.86 (0.53–1.38)	0.521
	Recessive	0.43 (0.17–1.08)	0.074	0.86 (0.47–1.57)	0.613	1.48 (0.27–8.11)	0.651	0.74 (0.44–1.23)	0.247	0.28 (0.10–0.79)	**0.015**
	Additive	0.85 (0.58–1.23)	0.374	0.92 (0.72–1.17)	0.488	0.82 (0.37–1.84)	0.628	0.89 (0.72–1.09)	0.259	0.74 (0.50–1.09)	0.124
rs4646440	Allele	0.70 (0.46–1.05)	0.086	0.90 (0.69–1.18)	0.447	3.83 (1.22–12.07)	**0.016**	0.87 (0.69–1.10)	0.254	0.87 (0.58–1.30)	0.495
	Codominant	0.24 (0.05–1.16)	0.075	0.40 (0.15–1.08)	0.071	–	–	0.39 (0.16–0.97)	**0.042**	0.50 (0.08–3.11)	0.453
		0.78 (0.47–1.28)	0.321	1.03 (0.75–1.42)	0.850	2.78 (0.71–10.84)	0.141	0.97 (0.73–1.28)	0.822	0.89 (0.54–1.46)	0.634
	Dominant	0.71 (0.43–1.16)	0.168	0.97 (0.71–1.32)	0.823	3.18 (0.84–12.00)	0.087	0.91 (0.70–1.20)	0.508	0.86 (0.53–1.41)	0.554
	Recessive	0.26 (0.05–1.26)	0.095	0.40 (0.15–1.06)	0.065			0.40 (0.16–0.97)	**0.043**	0.52 (0.08–3.22)	0.480
	Additive	0.67 (0.44–1.04)	0.074	0.90 (0.68–1.18)	0.438	3.15 (0.9–11.08)	0.073	0.86 (0.68–1.10)	0.225	0.85 (0.54–1.33)	0.467
rs35564277	Allele	0.65 (0.32–1.31)	0.223	0.84 (0.55–1.28)	0.407	1.08 (0.17–6.70)	0.938	0.80 (0.55–1.16)	0.239	0.66 (0.34–1.30)	0.227
	Codominant	–	–	–	–	–	–	–	–	–	–
		0.68 (0.32–1.44)	0.313	0.91 (0.58–1.43)	0.672	–	–	0.87 (0.59–1.30)	0.508	–	–
	Dominant	0.65 (0.31–1.36)	0.253	0.87 (0.55–1.36)	0.531	0.89 (0.13–6.10)	0.909	0.83 (0.56–1.23)	0.357	0.68 (0.33–1.39)	0.288
	Recessive	–	–	–	–	–	–	–	–	–	–
	Additive	0.63 (0.31–1.30)	0.211	0.83 (0.54–1.28)	0.402	0.89 (0.13–6.10)	0.909	0.80 (0.54–1.17)	0.240	0.68 (0.33–1.39)	0.288
rs4646437	Allele	1.21 (0.74–1.97)	0.450	0.73 (0.54–1.00)	**0.046**	0.36 (0.11–1.14)	0.074	0.80 (0.61–1.05)	0.111	0.97 (0.59–1.60)	0.918
	Codominant	0.59 (0.05–6.61)	0.666	0.14 (0.02–1.18)	0.071	–	–	0.12 (0.01–0.97)	**0.046**	–	–
		1.35 (0.78–2.34)	0.286	0.78 (0.55–1.11)	0.170	0.45 (0.12–1.68)	0.232	0.88 (0.65–1.19)	0.405	0.97 (0.55–1.70)	0.915
	Dominant	1.31 (0.76–2.24)	0.336	0.74 (0.53–1.05)	0.092	0.41 (0.11–1.51)	0.179	0.83 (0.62–1.12)	0.227	1.00 (0.57–1.75)	0.993
	Recessive	0.54 (0.05–6.10)	0.620	0.15 (0.02–1.26)	0.080	–	–	0.12 (0.02–1.00)	0.050	–	–
	Additive	1.23 (0.74–2.04)	0.430	0.72 (0.52–0.99)	**0.044**	0.41 (0.12–1.42)	0.159	0.79 (0.60–1.05)	0.103	1.04 (0.61–1.80)	0.876

*SNP, single nucleotide polymorphism; OR, odds ratio; CI, confidence interval. Bold data means significant difference (p < 0.05). –, No data*.

**Figure 1:**
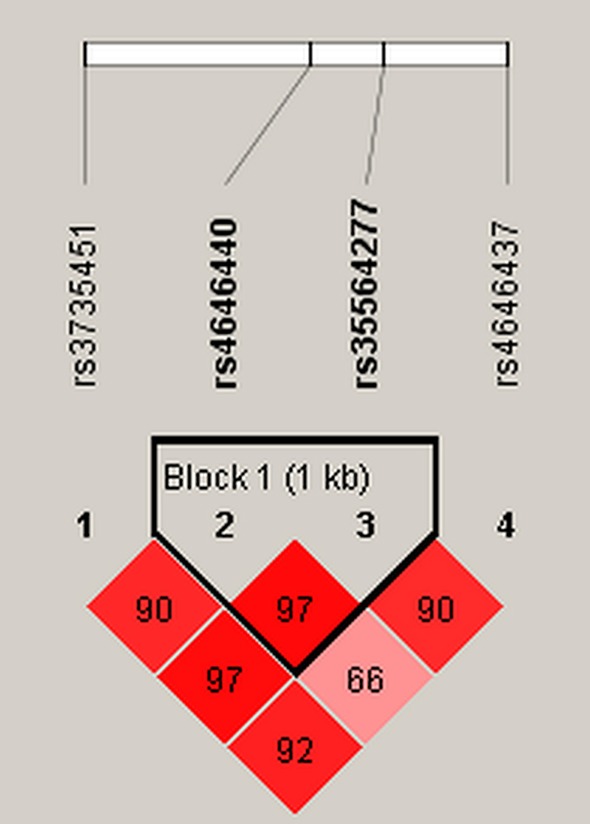
Haplotype block map for *CYP3A4* polymorphisms. Block 1 includes rs4646440 and rs35564277. The numbers inside the diamonds indicate the D′ for pairwise analyses.

And, we did haplotype analysis of association between *CYP3A4* polymorphisms and drug addiction risk (Table 5). We did not observe significant relationships between *CYP3A4* haplotype and drug addiction risk (*p* > 0.05). In Figure 1, we detected one block (rs4646440 and rs35564277).

**Table 5:** Haplotype analysis of association between *CYP3A4* polymorphisms and drug addiction risk.

**SNP**	**Haplotype**	**Frequency in cases**	**Frequency in controls**	**OR(95%CI)**	***p***	**OR(95%CI)**	***p***
rs4646440|rs35564277	AC	0.942	0.929	1.26 (0.87–1.80)	0.220	1.26 (0.87–1.81)	0.217
rs4646440|rs35564277	AT	0.859	0.855	1.03 (0.80–1.34)	0.800	1.04 (0.80–1.35)	0.780
rs4646440|rs35564277	GT	0.800	0.783	1.12 (0.89–1.40)	0.322	1.12 (0.90–1.41)	0.310

*SNP, single nucleotide polymorphism; OR, odds ratio; CI, confidence interval*.

## Discussion

In this study, we firstly found that *CYP3A4* polymorphisms (rs3735451, rs4646440, rs35564277, and rs4646437) were associated with drug addiction risk among the Chinese Han population. Especially, rs4646440 and rs4646437 were significantly associated with decreased risk of drug addiction. Stratified analysis showed that the effects of rs4646440 and rs4646437 on drug addiction risk are dependent on age and gender. Drug-using time also related to the association of rs3735451 and drug addiction risk. In addition, we observed one block (rs4646440 and rs35564277) by haplotype analysis.

*CYP3A4* is the major congener of CYP family, which is highly expressed in intestine and liver. *In vitro*, experiment showed morphine enhances *CYP3A4* expression (26). *CYP3A4* controls the metabolism of more than 70% of all drugs in human, including cocaine and opiate (27, 28). However, inter-individual variation in drug response is obvious. Growing evidence indicates that inter-individual variability in drug response is related to genetic polymorphisms. *CYP3A* isoenzymes are related to patients with alcohol use disorder by regulating haloperidol concentration (29).The effects of *CYP3A4* rs4646437 on drug is the most studied. He et al. found rs4646437 related to voriconazole metabolism, suggesting the impact of *CYP3A4* on the pharmacokinetics of antifungal agent (30). Among Chinese renal transplant recipients, rs4646437 could affect the interindividual variability in the metabolism of tacrolimus (31). In addition, rs4646437 was significantly associated with prostate cancer by modifying finasteride concentration (32). In this study, we observed rs4646440 and rs4646437 of *CYP3A4* had strong relationships with drug addiction risk. It suggests the role of *CYP3A4* polymorphisms in drug addiction. Further functional studies are needed to verify the effects of *CYP3A4* polymorphisms on drug addiction.

Although drug addiction generally occurs in the young, increasing prevalence drug use by elderly people is not ignorable (33). It was reported that drug addiction accelerated aging process in aging drug users (33). According to age classification criteria of the World Health Organization (WHO), we divided individuals into three groups (youth, middle-aged people and elderly people). In the subgroup of age ≥ 60, rs4646440 significantly increased risk of drug addiction. Rs4646437 was associated with drug addiction for the middle-aged people (44 <Age ≤ 59). Additionally, sex differences in drug abuse are common in drug addiction. For instance, men are more likely to use heroin than women and men take greater amounts of heroin (34, 35), it may be attributed to the molecular neuroadaptations. For participants in this study, there are more men than women. We hence performed association analysis in man and found *CYP3A4* polymorphisms (rs3735451, rs4646440, and rs4646437) decreased drug addiction risk. Finally, we explored the association of *CYP3A4* polymorphisms and drug addiction risk in individuals had different drug-using time. We observed that the influence of rs3735451 on drug addiction risk was related to drug-using time. Our results indicated that age, gender and drug-using time affect the relationship between *CYP3A4* polymorphisms and drug addiction risk. The exact mechanisms are required to study in the future.

Some limitations could not be ignored in this study. First, study participants are limited to the Chinese Han population. Second, drug addiction is caused by multiple factors, we could not eliminate the effects of all potential factors on drug addiction risk. Third, we could not do more stratified analyses due to limited information of participants or characteristic discrepancy. Hence, more ethnic population and well-designed studies are required to verify the association of *CYP3A4* polymorphisms and drug addiction risk.

## Conclusion

In summary, our study suggests that *CYP3A4* polymorphisms could be associated with drug addiction risk among the Chinese Han population and the associations are related to age, gender and drug-using time. Further studies in larger population with more experiments are required to confirm our results.

## Data Availability Statement

The raw data supporting the conclusions of this manuscript will be made available by the authors, without undue reservation, to any qualified researcher.

## Author Contributions

LW, MB, TJ, and JZ performed this study. YW, YH, and DY collected samples. XH supervised this study.

### Conflict of Interest

The authors declare that the research was conducted in the absence of any commercial or financial relationships that could be construed as a potential conflict of interest.
